# Identification of potential biomarkers and therapeutic targets for posttraumatic acute respiratory distress syndrome

**DOI:** 10.1186/s12920-023-01482-2

**Published:** 2023-03-14

**Authors:** Peng Qi, Mengjie Huang, Tanshi Li

**Affiliations:** 1 Department of Emergency, First Medical Center of Chinese PLA General Hospital, 28 Fuxing Road, Beijing, 100853 China; 2 Department of Nephrology, First Medical Center of Chinese PLA General Hospital, 28 Fuxing Road, Beijing, 100853 China

**Keywords:** Acute respiratory distress syndrome, Trauma, Bioinformatic analysis, Differentially expressed genes, Gene expression omnibus

## Abstract

**Background:**

Despite improved supportive care, posttraumatic acute respiratory distress syndrome (ARDS) mortality has improved very little in recent years. Additionally, ARDS diagnosis is delayed or missed in many patients. We analyzed co-differentially expressed genes (co-DEGs) to explore the relationships between severe trauma and ARDS to reveal potential biomarkers and therapeutic targets for posttraumatic ARDS.

**Methods:**

Two gene expression datasets (GSE64711 and GSE76293) were downloaded from the Gene Expression Omnibus. The GSE64711 dataset included a subset of 244 severely injured trauma patients and 21 healthy controls. GSE76293 specimens were collected from 12 patients with ARDS who were recruited from trauma intensive care units and 11 age- and sex-matched healthy volunteers. Trauma DEGs and ARDS DEGs were identified using the two datasets. Subsequently, Gene Ontology, Kyoto Encyclopedia of Genes and Genomes, and protein–protein interaction network analyses were performed to elucidate the molecular functions of the DEGs. Then, hub genes of the co-DEGs were identified. Finally, to explore whether posttraumatic ARDS and septic ARDS are common targets, we included a third dataset (GSE100159) for corresponding verification.

**Results:**

90 genes were upregulated and 48 genes were downregulated in the two datasets and were therefore named co-DEGs. These co-DEGs were significantly involved in multiple inflammation-, immunity- and neutrophil activation-related biological processes. Ten co-upregulated hub genes (GAPDH, MMP8, HGF, MAPK14, LCN2, CD163, ENO1, CD44, ARG1 and GADD45A) and five co-downregulated hub genes (HERC5, IFIT2, IFIT3, RSAD2 and IFIT1) may be considered potential biomarkers and therapeutic targets for posttraumatic ARDS. Through the verification of the third dataset, posttraumatic ARDS may have its own unique targets worthy of further exploration.

**Conclusion:**

This exploratory analysis supports a relationship between trauma and ARDS pathophysiology, specifically in relationship to the identified hub genes. These data may serve as potential biomarkers and therapeutic targets for posttraumatic ARDS.

## Background

Despite the continuous progress in the establishment of safety measures, trauma still accounts for more than one-tenth of deaths worldwide [1]. The burden is highest in individuals < 50years of age, among whom injury as a cause of death is second only to infectious diseases [2]. In addition to directly causing human tissue damage, trauma can cause a series of reactions both locally and systemically that lead to aggravation of the injury. The lung is the only organ that receives the entire cardiac output. In addition to being damaged by in situ-produced inflammatory mediators, it can be damaged by circulating inflammatory mediators produced by distant organs. Thus, lung injury occurs early, and severe injury can be caused by indirect traumatic factors. In 1967, Ashbaugh described 12 patients, including 7 patients with severe trauma who appeared to have acute hypoxemia, noncardiogenic pulmonary edema, reduced lung compliance (increased lung stiffness), increased work of breathing and the need for positive-pressure ventilation [3]. This series of syndromes was first named adult respiratory distress syndrome. It was subsequently renamed acute respiratory distress syndrome (ARDS) in a number of studies. Globally, ARDS affects approximately 3 million patients annually and accounts for 10% of intensive care unit (ICU) admissions, and 24% of patients with ARDS receive mechanical ventilation in the ICU [4]. In patients with traumatic injuries, inflammatory responses at the local and systemic levels affect the lung both directly and indirectly and are the common cause of ARDS. Approximately 5–10% of adult trauma patients develop ARDS [5], and up to 19% of these patients are admitted to the ICU. Studies of patients with posttraumatic ARDS have identified a mortality of between 16 and 24% [6], and the mortality of severely injured trauma patients with ARDS can reach 35–45% [7]. Despite decades of research and considerable advances in our understanding of the pathogenesis, risk factors and complication management of ARDS, there has been no change in the mortality rate of posttraumatic ARDS over the last four decades [8]. This observation suggests that posttraumatic ARDS has properties distinct from those of other forms of ARDS. Furthermore, although the definition and diagnosis of ARDS have been continuously improved, clinicians miss the diagnosis of 40% of ARDS cases [8] because the assessment and diagnosis of ARDS are operator-dependent and partially subjective, leading to high interobserver variability and compromising diagnostic accuracy. In recent years, great effort has been dedicated to identifying biomarkers of ARDS. Precise diagnostic biomarkers and biomarkers that suggest the severity of ARDS may improve early diagnosis [9]. However, at present, diagnosis is still made in the absence of established biomarkers [10].

To identify and develop precise diagnostic biomarkers and therapeutic strategies for posttraumatic ARDS, a better understanding of the mechanisms leading to lung damage as well as recovery in trauma patients is essential. In our study, two datasets were downloaded from the Gene Expression Omnibus (GEO) and analyzed to identify co-differentially expressed genes (co-DEGs) associated with severe injury and ARDS. Then, we elucidated the molecular mechanisms of trauma-related DEGs and ARDS-related DEGs through functional and pathway analyses and protein–protein interaction (PPI) network analysis. Then, we screened potential hub genes and refer to common public databases for analysis one by one.Finally, since previous studies on ARDS were mostly focused on sepsis, there were few studies on posttraumatic ARDS targets. It is unclear whether they have a common pathway or a special target. However, the latest research suggests that the mortality rate of post-traumatic ARDS has not decreased due to the improvement of medical technology, but has increased [11]. Based on this, we introduced the third sepsis related ARDS database for parallel verification to explore whether the ARDS caused by the two causes is caused by a common target, so as to verify whether the specificity of posttraumatic ARDS is worthy of further independent research.

## Materials and methods

### Microarray data

Microarray studies in this paper were searched from the GEO database [12] (https://www.ncbi.nlm.nih.gov/geo/) using the terms “acute respiratory distress syndrome” and “trauma”. Two datasets (GSE64711 [13] and GSE76293 [14]) were selected for subsequent analysis. We used the “GEOquery” package [15] of R software (version 4.0.2, http://r-project.org/) to download the expression profile datasets GSE64711 and GSE76293 from the GEO database. The two datasets included all necessary information, and no samples had to be taken on site. The GSE64711 dataset included a subset of 244 severely injured trauma patients aged 16 to 90 years old, and an additional 21 healthy controls were enrolled for blood sampling for enriched polymorphonuclear neutrophil genomic analysis. Among them, the specimen collection time of trauma patients is within 4 days after the trauma, which also meets the requirements of the Berlin standard of ARDS. The inclusion criteria of GSE64711 included adult patients (age ≥ 16 years) who had been severely injured (injury severity score (ISS) > 15) after having undergone blunt trauma without severe traumatic brain injury (TBI) and with evidence of hemorrhagic shock (systolic blood pressure (SBP) < 90 mmHg or base deficit ≥ 6 mEq/L and requiring blood transfusion). Specimens of GSE76293 were collected from 12 patients with ARDS who were recruited from trauma ICUs in a U.K. teaching hospital and from 11 age- and sex-matched healthy volunteers. GSE64711 was profiled on a GPL19607 [hGlue1_0.r3] custom Affymetrix Human Transcriptome Array, and GSE76293 was profiled on a GPL570 [HG-U133_Plus_2] Affymetrix Human Genome U133 Plus 2.0 Array. All RNA information of the selected samples was downloaded and the original microarray datasets were normalized and preprocessed with R software.

### Identification of DEGs

We used the “limma” [16] package of R software to screen DEGs. A p value of < 0.05 and an absolute value of the log2 (fold change) of > 1 were the thresholds for identifying differences in gene expression as significant [16, 17]. Volcano plots were generated using the R package “ggplot2” [18].

### Gene ontology (GO) and Kyoto encyclopedia of genes and genomes (KEGG) pathway enrichment analyses of DEGs

DEGs from the two datasets were screened for subsequent GO and KEGG pathway enrichment analyses. GO function analysis (with the cellular component [CC], biological process [BP], and molecular function [MF] categories) is a powerful bioinformatics tool to classify gene expression and its properties [19]. KEGG pathway analysis was used to identify the cell pathways in which the DEGs might be involved [20]. GO and KEGG pathway enrichment analyses were performed using the clusterProfiler [21] routine in R. P < 0.05 was considered statistically significant.

### PPI network construction and hub gene identification

We used the “VennDiagram” [22] package of R software to screen the co-upregulated and co-downregulated genes in the two datasets, which were constructed using the Search Tool for the Retrieval of Interacting Genes (STRING; http://string-db.org/) [23]. A minimum required interaction score > 0.4 was set as the cutoff point. Subsequently, Cytoscape software was used to construct and visualize molecular interaction networks. The hub genes in the PPI networks were selected using the Cyto-Hubba plug-in of Cytoscape.

### Functional enrichment analysis of hub genes

The selected hub genes were analyzed by the PANTHER [24] classification system (http://pantherdb.org/), and the basic classification of each gene was obtained. Then, the Human Protein Atlas [25] (HPA) (https://www.proteinatlas.org/) was used to explore the expression profile of each hub gene in human tissue. Subsequently, the Deeply Integrated human Single-Cell Omics (DISCO) database [26] (https://www.immunesinglecell.org/) was used to clarify the expression level of the hub genes in lung. Finally, the “clusterProfiler” [21] package of R software was used for the enrichment analysis of the hub genes, and the “ggplot2” package of R software was used to visually present the results of the enrichment analysis. The results are displayed in a bubble plot and table.

### Verification of hub genes

To explore whether posttraumatic ARDS and septic ARDS are common targets, or posttraumatic ARDS has its unique pathways and targets. We included a third data set for corresponding verification. We searched the GEO database with the keyword “sepsis”. Then searched using keywords and restricted the screening conditions to “Homo sapiens” and “Expression profiling by array” and limited the sample to peripheral blood. After reading the original studies on the data sets that met the screening conditions individually, we focused on the experimental design and methodology. The data set that met the principle of randomized control and did not violate ethical guidelines was taken as the research material.

## Results

### Data details and preprocessing

We analyzed peripheral blood samples from three independent GEO datasets. A total of 244 severely injured trauma patients, 12 patients with ARDS, 35 patients with sepsis and 44 healthy controls were included in this study. Ethical approval was not necessary because our study was a bioinformatic analysis.

### Identification of DEGs

**Fig. 1:**
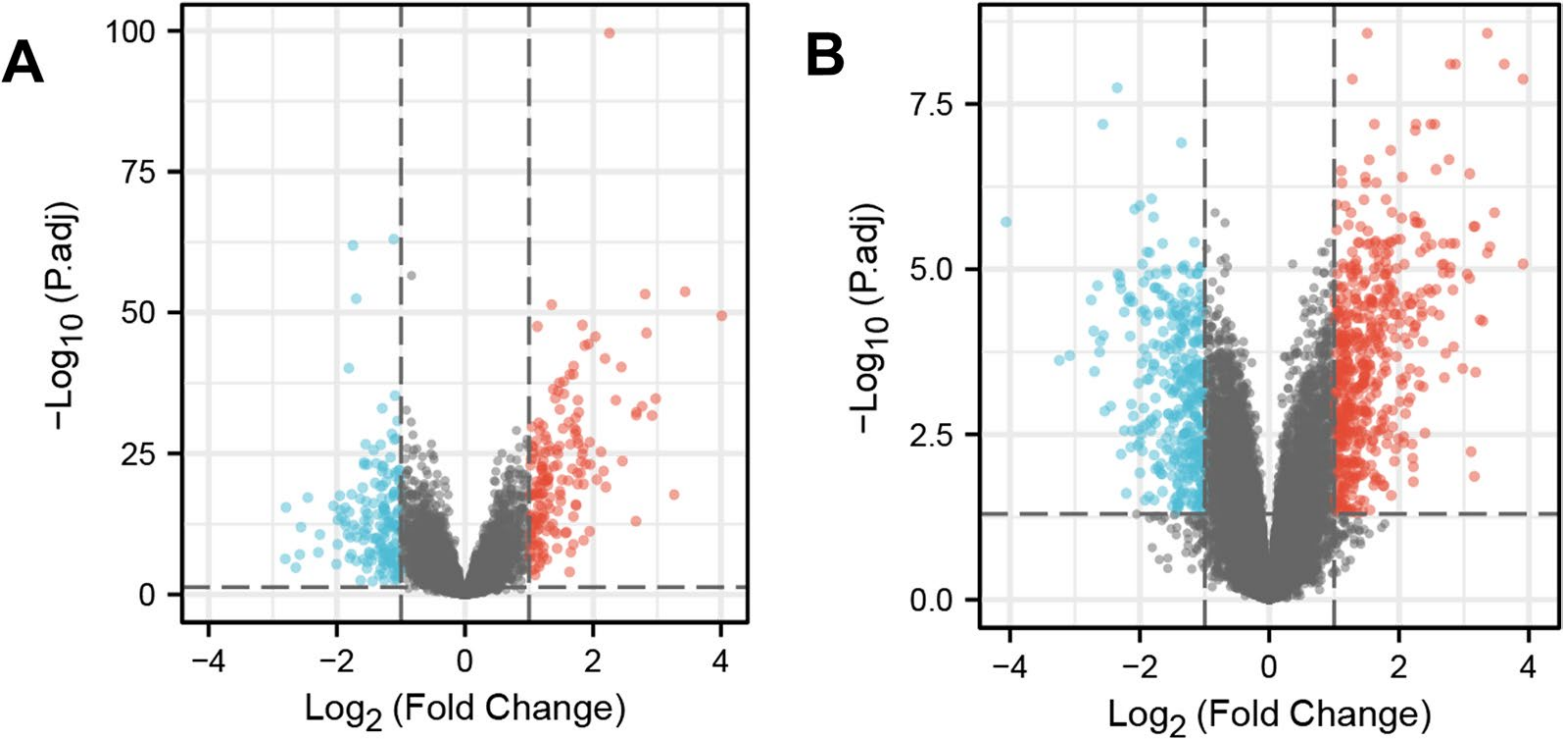
DEGs in the two datasets. **A** Volcano plot of GSE64711, red represents upregulated genes, blue represents downregulated genes, and gray represents no significantly expressed genes; **B** Volcano plot of GSE76293. The criteria for statistically significant difference of DEGs was adjusted |log2(FC)|> 1 and p.adj < 0.05 in expression

The “limma” package of R software was used to screen DEGs in the three datasets. In the trauma dataset, the total number of IDs after removing the null values was 19,844, of which 337 IDs met the threshold of |log2(FC)|> 1 and p.adj < 0.05. The number of upregulated DEGs (log2(FC) > 1) was 172, and the number of downregulated DEGs (log2(FC) < − 1) was 165. In the ARDS dataset, the total number of IDs after removing the null values was 45,118, of which 894 IDs met the threshold of |log2(FC)|> 1 and p.adj < 0.05. The number of upregulated DEGs (log2(FC) > 1) was 541, and the number of downregulated DEGs (log2(FC) < − 1) was 353. The DEGs were visualized using the “ggplot2” package (Fig. 1).

### GO and KEGG pathway enrichment analyses of DEGs

**Fig. 2:**
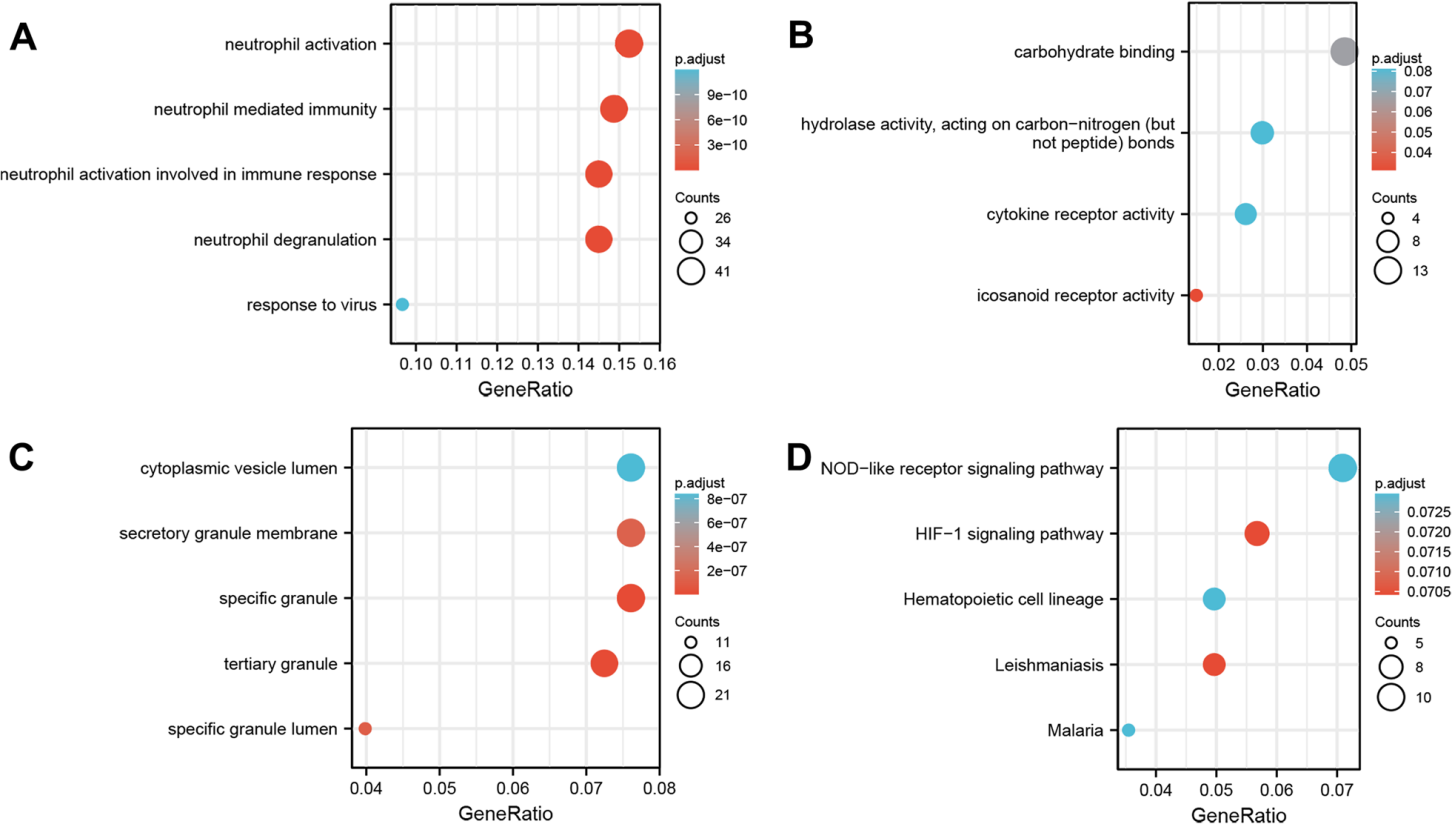
GO and KEGG enrichment analysis of DEGs in GSE64711. **A** Shows the results of biological process terms enriched by BP analysis; **B** Shows the results of biological process terms enriched by MF; **C** Shows the results of biological process terms enriched by CC analysis; **D** Shows the enriched pathway by KEGG analysis. The coloured dots represent the P-value for that term, with red representing greater significance. The size of the dots represents the number of involved genes

**Fig. 3:**
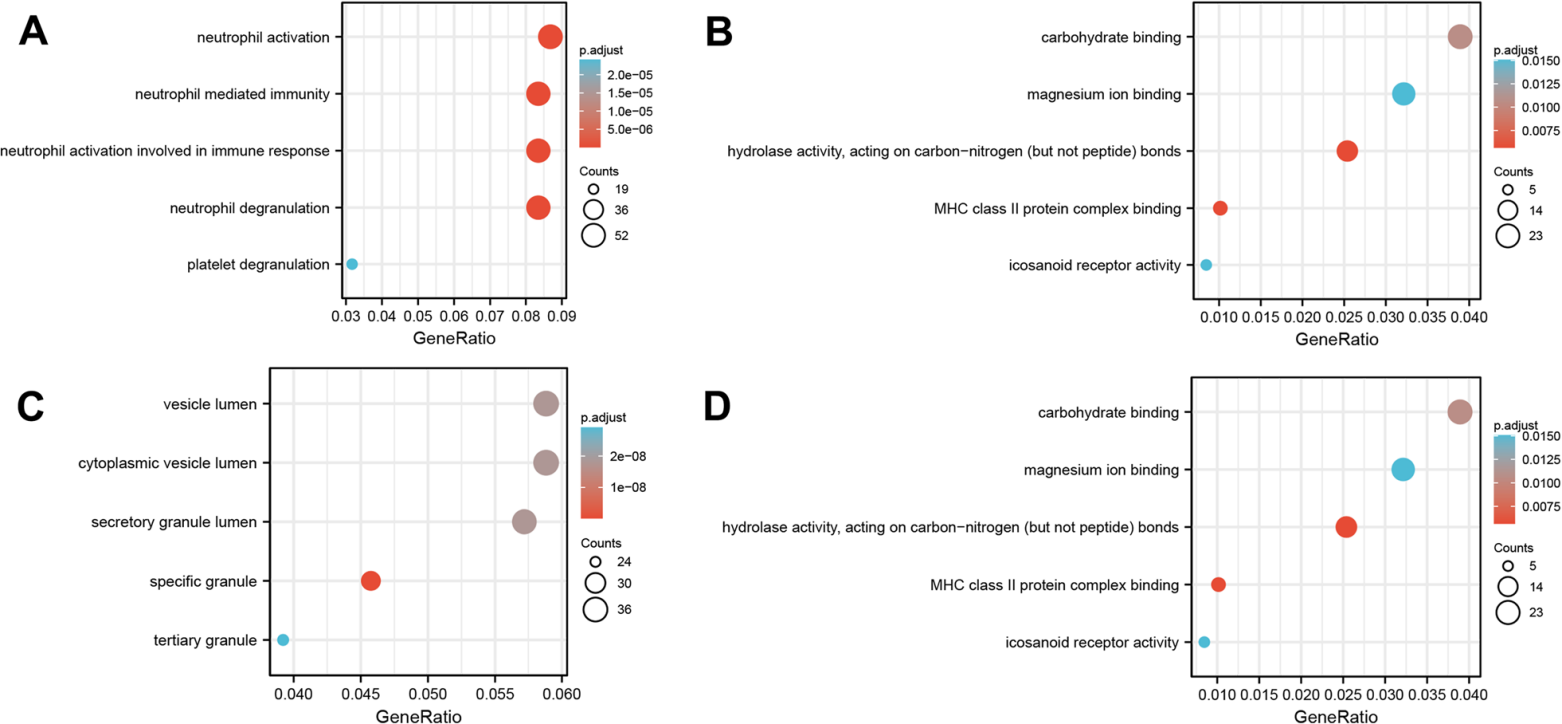
GO and KEGG enrichment analysis of DEGs in GSE76293. **A** Shows the results of biological process terms enriched by BP analysis; **B** Shows the results of biological process terms enriched by MF; **C** Shows the results of biological process terms enriched by CC analysis; **D** Shows the enriched pathway by KEGG analysis

GO and KEGG pathway enrichment analyses of the DEGs of GSE64711 and GSE76293 were performed using the clusterProfiler routine in R, and enrichment analysis results were visualized using the R package “ggplot2” (Figs. 2, 3). The significantly enriched CC, BP, and MF terms and metabolic pathways of the DEGs of severe injury trauma and posttraumatic ARDS are shown (Tables 1, 2).

**Table 1:** Details for GO terms and KEGG pathway enrichment in GSE64711

Ontology	ID	Description	GeneRatio	BgRatio	pvalue	p.adjust	qvalue
BP	GO:0042119	Neutrophil activation	41/269	498/18670	1.10e−19	3.90e−16	3.15e−16
BP	GO:0002446	Neutrophil mediated immunity	40/269	499/18670	8.35e−19	1.48e−15	1.19e−15
BP	GO:0043312	Neutrophil degranulation	39/269	485/18670	2.13e−18	2.34e−15	1.89e−15
CC	GO:0042581	Specific granule	21/276	160/19717	9.19e−15	3.20e−12	2.91e−12
CC	GO:0070820	Tertiary granule	20/276	164/19717	1.68e−13	2.93e−11	2.66e−11
CC	GO:0035580	Specific granule lumen	11/276	62/19717	8.95e−10	1.04e−07	9.46e−08
MF	GO:0004953	Icosanoid receptor activity	4/268	15/17697	6.15e−05	0.031	0.029
MF	GO:0030246	Carbohydrate binding	13/268	271/17697	2.63e−04	0.067	0.061
MF	GO:0016810	Hydrolase activity, acting on carbon–nitrogen (but not peptide) bonds	8/268	123/17697	5.72e−04	0.081	0.074
KEGG	hsa05140	Leishmaniasis	7/141	77/8076	3.70e−04	0.070	0.063
KEGG	hsa04066	HIF-1 signaling pathway	8/141	109/8076	6.10e−04	0.070	0.063
KEGG	hsa04621	NOD-like receptor signaling pathway	10/141	181/8076	0.001	0.073	0.066

**Table 2:** Details for GO terms and KEGG pathway enrichment in GSE76293

Ontology	ID	Description	GeneRatio	BgRatio	pvalue	p.adjust	qvalue
BP	GO:0042119	Neutrophil activation	21/86	498/18670	6.74e−15	1.27e−11	1.05e−11
BP	GO:0043312	Neutrophil degranulation	20/86	485/18670	4.98e−14	3.52e−11	2.89e−11
BP	GO:0002283	Neutrophil activation involved in immune response	20/86	488/18670	5.59e−14	3.52e−11	2.89e−11
BP	GO:0002446	Neutrophil mediated immunity	20/86	499/18670	8.48e−14	4.01e−11	3.29e−11
BP	GO:0001819	Positive regulation of cytokine production	10/86	464/18670	5.34e−05	0.018	0.015

**Fig. 4:**
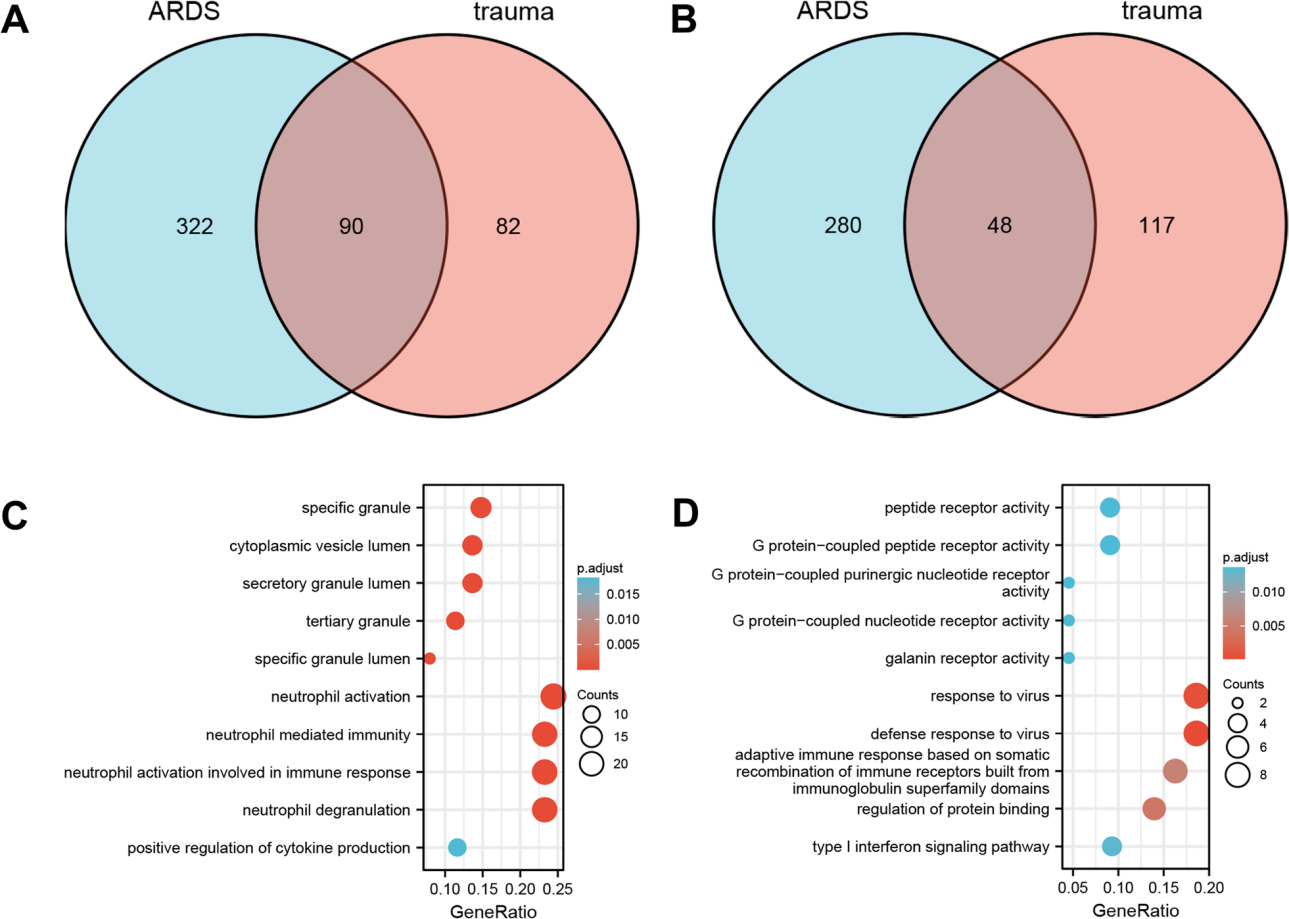
Venn diagrams and GO term enrichment analysis on common DEGs. **A** 90 co-upregulated DEGs were screened from the two datasets; **B** 48 co-downregulated DEGs were screened from the two datasets; **C** Shows the results of GO term enrichment analysis perform on the 90 co-upregulated DEGs; **D** Shows the results of GO term enrichment analysis perform on the 48 down-upregulated DEGs

The “VennDiagram” package of R was used to screen 90 co-upregulated DEGs and 48 co-downregulated DEGs from the two datasets (Fig. 4A, B). GO term enrichment analysis was performed on the common DEGs (Fig. 4C, D), and the results showed that the BPs of co-upregulated DEGs were mainly related to neutrophil activation, neutrophil degranulation, neutrophil activation involved in the immune response, neutrophil-mediated immunity and positive regulation of cytokine production, These BP processes are also the current focus of intense attention in ARDS. At the same time, we tabulated the results of the enrichment analysis of co-upregulated differential genes and co-downregulated differential genes in the form of a third line table (Tables 3, 4).

**Table 3:** Details for co-upregulated DEGs of GO terms enrichment in GSE64711 and GSE76293

Ontology	ID	Description	GeneRatio	BgRatio	pvalue	p.adjust	qvalue
BP	GO:0042119	Neutrophil activation	21/86	498/18670	6.74e−15	1.27e−11	1.05e−11
BP	GO:0043312	Neutrophil degranulation	20/86	485/18670	4.98e−14	3.52e−11	2.89e−11
BP	GO:0002283	Neutrophil activation involved in immune response	20/86	488/18670	5.59e−14	3.52e−11	2.89e−11
BP	GO:0002446	Neutrophil mediated immunity	20/86	499/18670	8.48e−14	4.01e−11	3.29e−11
BP	GO:0001819	Positive regulation of cytokine production	10/86	464/18670	5.34e−05	0.018	0.015
CC	GO:0042581	Specific granule	13/88	160/19717	2.87e−13	4.36e−11	3.75e−11
CC	GO:0070820	Tertiary granule	10/88	164/19717	3.11e−09	2.36e−07	2.03e−07
CC	GO:0035580	Specific granule lumen	7/88	62/19717	1.12e−08	5.65e−07	4.85e−07
CC	GO:0034774	Secretory granule lumen	12/88	321/19717	1.92e−08	7.29e−07	6.26e−07
CC	GO:0060205	Cytoplasmic vesicle lumen	12/88	338/19717	3.39e−08	8.87e−07	7.62e−07

**Table 4:** Details for co-downregulated DEGs of GO terms enrichment in GSE64711 and GSE76293

Ontology	ID	Description	GeneRatio	BgRatio	pvalue	p.adjust	qvalue
BP	GO:0051607	Defense response to virus	8/43	238/18670	6.12e−08	8.26e−05	6.81e−05
BP	GO:0009615	Response to virus	8/43	326/18670	6.76e−07	4.56e−04	3.76e−04
BP	GO:0043393	Regulation of protein binding	6/43	217/18670	9.80e−06	0.004	0.004
BP	GO:0002460	Adaptive immune response based on somatic recombination of immune receptors built from immunoglobulin superfamily domains	7/43	361/18670	1.69e−05	0.006	0.005
BP	GO:0060337	Type I interferon signaling pathway	4/43	95/18670	6.67e−05	0.013	0.011
MF	GO:0004966	Galanin receptor activity	2/44	10/17697	2.68e−04	0.014	0.011
MF	GO:0001608	G protein-coupled nucleotide receptor activity	2/44	13/17697	4.63e−04	0.014	0.011
MF	GO:0045028	G protein-coupled purinergic nucleotide receptor activity	2/44	13/17697	4.63e−04	0.014	0.011
MF	GO:0008528	G protein-coupled peptide receptor activity	4/44	146/17697	4.67e−04	0.014	0.011
MF	GO:0001653	Peptide receptor activity	4/44	152/17697	5.44e−04	0.014	0.011

### PPI network construction and hub gene identification

**Fig. 5:**
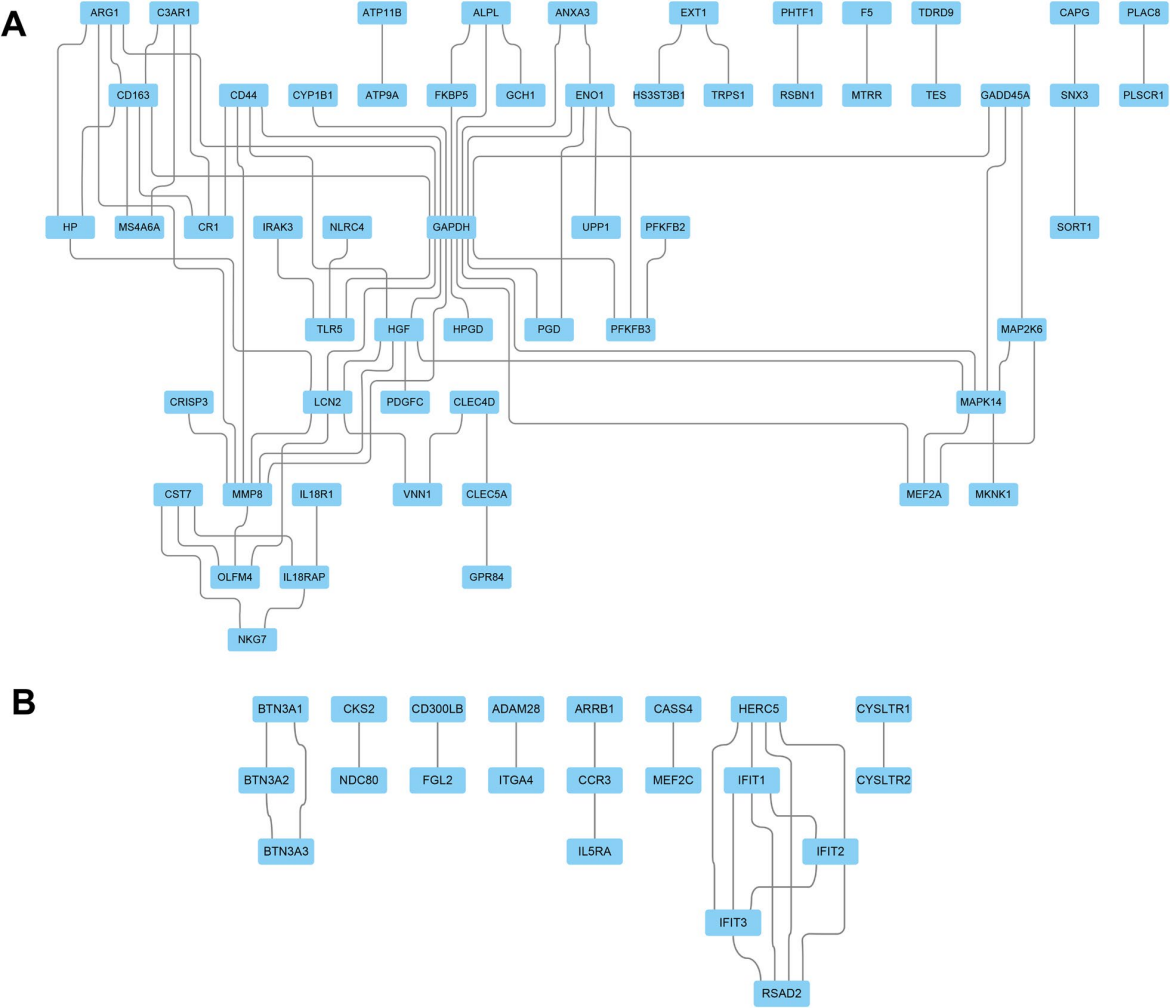
PPI network construction **A** PPI network of the co-upregulated DEGs. Each node stands for a gene or a protein, and edges represent the interactions between the nodes; **B** PPI network of the down-upregulated DEGs

The PPI network was constructed with the co-upregulated DEGs and co-downregulated DEGs, visualized and analyzed with Cytoscape software. Based on the identified DEGs, we constructed a PPI network for co-upregulated DEGs consisting of 57 nodes and 72 edges (Fig. 5A) and a PPI network for co-downregulated DEGs consissting of 21 nodes and 20 edges (Fig. 5B). The hub genes were selected by the maximal clique centrality (MCC) algorithm with the Cyto-Hubba plug-in of Cytoscape software and included glyceraldehyde-3-phosphate dehydrogenase (GAPDH), matrix metallopeptidase 8 (MMP8), hepatocyte growth factor (HGF), mitogen-activated protein kinase 14 (MAPK14), lipocalin 2 (LCN2), CD163 molecule (CD163), enolase 1 (EN01), CD44 molecule (CD44), arginase 1 (ARG1), and growth arrest and DNA-damage-inducible protein 45 alpha (GADD45A) which were co-upregulated hub DEGs. Besides, HECT and RLD domain containing E3 ubiquitin protein ligase 5 (HERC5), Interferon induced protein with tetratricopeptide repeats 2(IFIT2), Interferon induced protein with tetratricopeptide repeats 3(IFIT3), Radical S-adenosyl methionine domain containing 2(RSAD2), Interferon induced protein with tetratricopeptide repeats 1(IFIT1) were co-downregulated hub genes.

### Analysis of hub genes

**Fig. 6:**
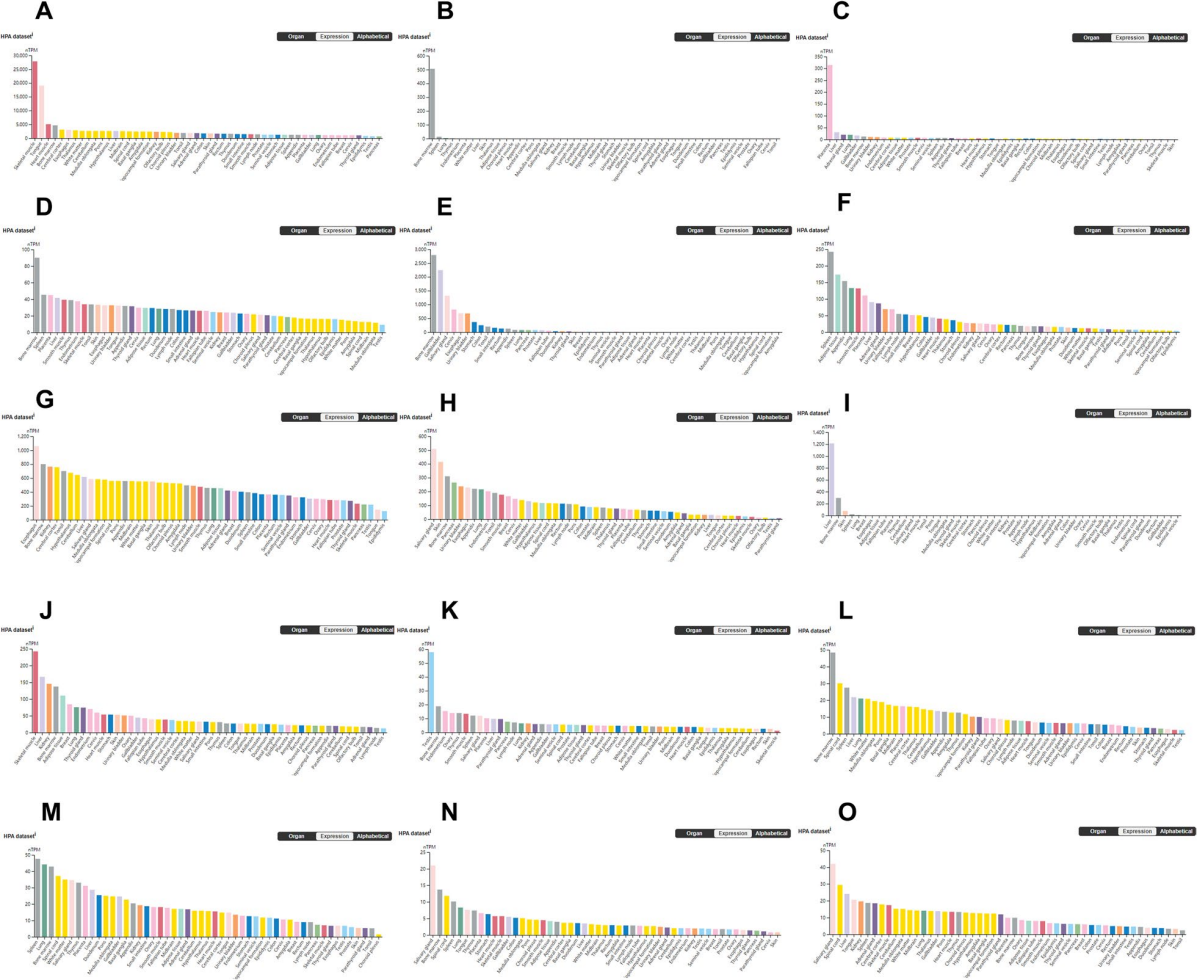
RNA expression overview shows RNA-seq tissue data from internally generated Human Protein Atlas (HPA) data. Color-coding is based on tissue groups, each consisting of tissues with functional features in common. **A** GAPDH **B** MMP8 **C** HGF **D** MAPK14 **E** LCN2 **F** CD163 **G** ENO1 **H** CD44 **I** ARG1 **J** GADD45A **K** HERC5 **L** IFIT2 **M** IFIT3 **N** RSAD2 **O** IFIT1

**Fig. 7:**
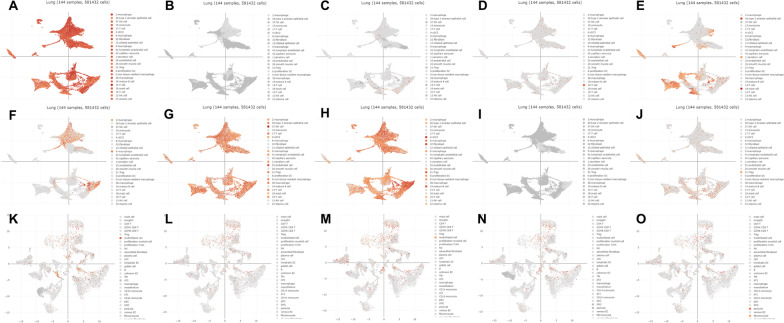
The DISCO database was used annotate the single-cell sequencing data of cells from lung. The deeper the color is, the higher the expression of the gene in the cell. **A** GAPDH **B** MMP8 **C** HGF **D** MAPK14 **E** LCN2 **F** CD163 **G** ENO1 **H** CD44 **I** ARG1 **J** GADD45A **K** HERC5 **L** IFIT2 **M** IFIT3 **N** RSAD2 **O** IFIT1

The PANTHER [24] classification system was used to analyze the selected hub genes, and the basic classification of each gene was obtained. The results are listed in a table (Table 5). To explore the expression of the hub genes in human tissue, the HPA database was used to analyze each hub gene (Fig. 6). The HPA RNA-seq tissue data is reported as nTPM (normalized protein-coding transcripts per million), corresponding to mean values of the different individual samples from each tissue. Color-coding is based on tissue groups, each consisting of tissues with functional features in common [25]. The DISCO [26] database was used annotate the single-cell sequencing data of cells from lung. The expression level of each hub gene in each cell is shown. The deeper the color is, the higher the expression of the gene in the cell (Fig. 7).

**Table 5:** The PANTHER classification system of hub genes

ID	Gene name	Gene ID	PANTHER family/subfamily	PANTHER protein class
ENO1	Alpha-enolase ENO1 ortholog	HUMAN|HGNC = 3350|UniProtKB = P06733	Alpha-enolase (PTHR11902:SF12)	Lyase
MMP8	Neutrophil collagenase MMP8 ortholog	HUMAN|HGNC = 7175|UniProtKB = P22894	Neutrophil collagenase (PTHR10201:SF137)	Metalloprotease
CD44	CD44 antigen CD44 ortholog	HUMAN|HGNC = 1681|UniProtKB = P16070	CD44 antigen (PTHR10225:SF6)	Transmembrane signal receptor
HGF	Hepatocyte growth factor HGF ortholog	HUMAN|HGNC = 4893|UniProtKB = P14210	Hepatocyte growth factor (PTHR24261:SF8)	Serine protease
GADD45A	Growth arrest and DNA damage-inducible protein GADD45 alpha GADD45A ortholog	HUMAN|HGNC = 4095|UniProtKB = P24522	Growth arrest and DNA damage-inducible protein GADD45 alpha (PTHR10411:SF3)	–
MAPK14	Mitogen-activated protein kinase 14 MAPK14 ortholog	HUMAN|HGNC = 6876|UniProtKB = Q16539	Mitogen-activated protein kinase 14 (PTHR24055:SF110)	Non-receptor serine/threonine protein kinase
CD163	Scavenger receptor cysteine-rich type 1 protein M130 CD163 ortholog	HUMAN|HGNC = 1631|UniProtKB = Q86VB7	Scavenger receptor cysteine-rich type 1 protein M130 (PTHR19331:SF441)	Serine protease
LCN2	Neutrophil gelatinase-associated lipocalin LCN2 ortholog	HUMAN|HGNC = 6526|UniProtKB = P80188	Neutrophil gelatinase-associated lipocalin (PTHR11430:SF13)	Transfer/carrier protein
GAPDH	Glyceraldehyde-3-phosphate dehydrogenase GAPDH ortholog	HUMAN|HGNC = 4141|UniProtKB = P04406	Glyceraldehyde-3-phosphate dehydrogenase (PTHR10836:SF111)	Dehydrogenase
ARG1	Arginase-1 ARG1 ortholog	HUMAN|HGNC = 663|UniProtKB = P05089	Arginase-1 (PTHR43782:SF2)	Hydrolase
HERC5	E3 ISG15–protein ligase HERC5HERC5	HUMAN|HGNC = 24368|UniProtKB = Q9UII4	E3 ISG15–protein ligase HERC5 (PTHR45622:SF7)	Ubiquitin-protein ligase
IFIT2	Interferon-induced protein with tetratricopeptide repeats 2 IFIT2	HUMAN|HGNC = 5409|UniProtKB = P09913	Interferon-induced protein with tetratricopeptide repeats 2 (PTHR10271:SF4)	Defense/immunity protein
IFIT3	Interferon-induced protein with tetratricopeptide repeats 3 IFIT3	HUMAN|HGNC = 5411|UniProtKB = O14879	Interferon-induced protein with tetratricopeptide repeats 3 (PTHR10271:SF3)	Defense/immunity protein
RSAD2	Radical S-adenosyl methionine domain-containing protein 2 RSAD2	HUMAN|HGNC = 30908|UniProtKB = Q8WXG1	Radical S-adenosyl methionine domain-containing protein 2 (PTHR21339:SF0)	–
IFIT1	Interferon-induced protein with tetratricopeptide repeats 1 IFIT1	HUMAN|HGNC = 5407|UniProtKB = P09914	Interferon-induced protein with tetratricopeptide repeats 1 (PTHR10271:SF16)	Defense/immunity protein

### Functional enrichment analysis of hub genes

**Fig. 8:**
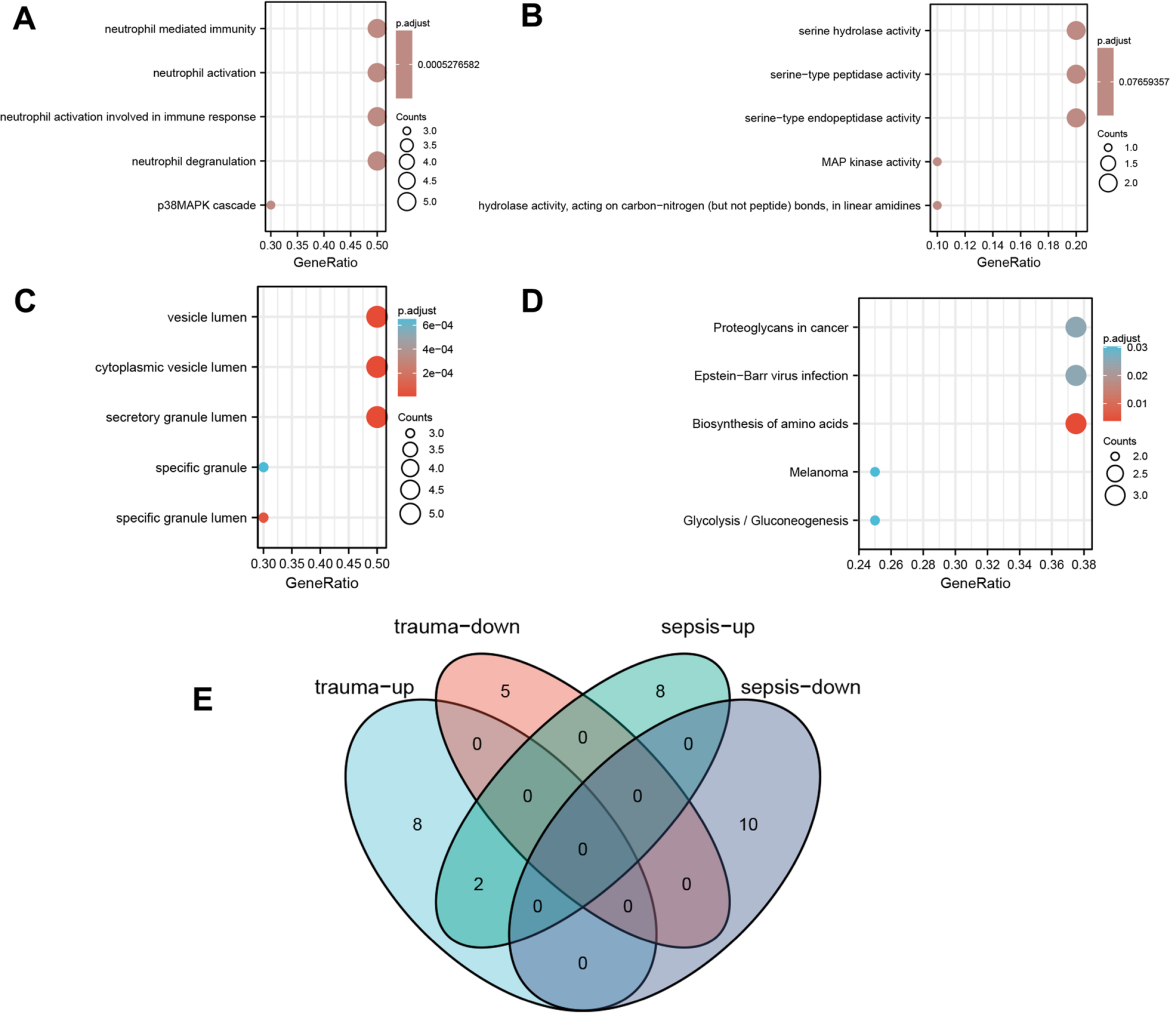
Functional enrichment analysis of the ten co-upregulated hub genes and the Venn diagrams. **A** Shows the results of biological process terms enriched by BP analysis; **B** Shows the results of biological process terms enriched by MF analysis; **C** Shows the results of biological process terms enriched by CC analysis; **D** Shows the enriched pathway by KEGG analysis; **E** the Venn diagrams between the posttraumatic ARDS and the septic ARDS groups in hub genes

Since the up-regulated DEG is a risk factor for the disease group, it is of greater significance for the occurrence and development of the disease. Therefore, we once again performed enrichment analysis on the co-upregulated hub genes to find out the commonness of this group of specific gene sets in biological composition / function / process. GO and KEGG pathway enrichment analyses of the ten co-upregulated hub genes (GAPDH, MMP8, HGF, MAPK14, LCN2, CD163, ENO1, CD44, ARG1 and GADD45A) were performed and the results showed that the main enriched BP terms were the neutrophil mediated immunity, neutrophil activation, neutrophil activation involved in immune response and neutrophil degranulation. The main enriched CC terms were the secretory granule lumen, cytoplasmic vesicle lumen and vesicle lumen, and the main enriched MF term were the serine-type endopeptidase activity, serine-type peptidase activity and serine hydrolase activity. The KEGG pathway analysis mainly showed enrichment of the terms biosynthesis of amino acids, Epstein–Barr virus infection and proteoglycans in cancer. All results are displayed in a bubble plot (Fig. 8A–D) and table (Table 6).

**Table 6:** Details of GO terms and KEGG pathway enrichment in co-upregulated hub genes

Ontology	ID	Description	GeneRatio	BgRatio	pvalue	p.adjust	qvalue
BP	GO:0038066	p38MAPK cascade	3/10	53/18670	2.56e−06	5.28e−04	2.04e−04
BP	GO:0043312	Neutrophil degranulation	5/10	485/18670	2.62e−06	5.28e−04	2.04e−04
BP	GO:0002283	Neutrophil activation involved in immune response	5/10	488/18670	2.70e−06	5.28e−04	2.04e−04
BP	GO:0042119	Neutrophil activation	5/10	498/18670	2.99e−06	5.28e−04	2.04e−04
BP	GO:0002446	Neutrophil mediated immunity	5/10	499/18670	3.02e−06	5.28e−04	2.04e−04
CC	GO:0034774	Secretory granule lumen	5/10	321/19717	2.61e−07	6.17e−06	4.57e−06
CC	GO:0060205	Cytoplasmic vesicle lumen	5/10	338/19717	3.38e−07	6.17e−06	4.57e−06
CC	GO:0031983	Vesicle lumen	5/10	339/19717	3.43e−07	6.17e−06	4.57e−06
CC	GO:0035580	Specific granule lumen	3/10	62/19717	3.50e−06	4.72e−05	3.50e−05
CC	GO:0042581	Specific granule	3/10	160/19717	6.04e−05	6.52e−04	4.83e−04
MF	GO:0004252	Serine-type endopeptidase activity	2/10	160/17697	0.003	0.077	0.042
MF	GO:0008236	Serine-type peptidase activity	2/10	182/17697	0.004	0.077	0.042
MF	GO:0017171	Serine hydrolase activity	2/10	186/17697	0.005	0.077	0.042
MF	GO:0016813	Hydrolase activity, acting on carbon–nitrogen (but not peptide) bonds, in linear amidines	1/10	11/17697	0.006	0.077	0.042
MF	GO:0004707	MAP kinase activity	1/10	14/17697	0.008	0.077	0.042
KEGG	hsa01230	Biosynthesis of amino acids	3/8	75/8076	4.17e−05	0.004	0.003
KEGG	hsa05169	Epstein–Barr virus infection	3/8	202/8076	7.87e−04	0.025	0.020
KEGG	hsa05205	Proteoglycans in cancer	3/8	205/8076	8.21e−04	0.025	0.020
KEGG	hsa00010	Glycolysis/gluconeogenesis	2/8	67/8076	0.002	0.030	0.024
KEGG	hsa05218	Melanoma	2/8	72/8076	0.002	0.030	0.024

### Verification of hub genes

Through conditional screening, we ultimately selected GSE100159 as the research object. The GSE100159 data set includes 35 sepsis patients and 12 healthy controls. The chip platform used is a GPL6884 Illumina HumanWG-6 v3.0 expression beadchip. Using the aforementioned methodology, we performed background correction and data normalization on the GSE100159 data set and then used the "limma" package of R software to analyze the disease group and the healthy control group to obtain DEGs. The inclusion criteria for DEGs were the same as those for the above two data sets. The DEGs of the GSE100159 and GSE76293 data sets were merged and analyzed, the co-DEGs were screened out, a PPI network was constructed, and the hub genes were extracted by Cytoscape software. The final results showed that the hub genes in the two data sets were FCGR1A, MPO, CR1, CEACAM8, CD163, ITGA2B, ITGB3, CD44, THBS1, PKM, RPL18A, RPL19, RPL8, RPL30, RPL18, RPL17, RPL27, RPL13, RPL10A and RPL22. It can be seen from the results that the hub genes of trauma and ARDS are not completely the same as those of sepsis and ARDS (Fig. 8E), which on the other hand shows that posttraumatic ARDS has its own characteristic targets and is worthy of further exploration.

## Discussion

Trauma associated with severe injury not only leads to direct damage in patients but also causes a secondary systemic inflammatory response, which could result in systemic inflammatory response syndrome and is strongly correlated with the development of severe posttraumatic multiple organ dysfunction syndrome (MODS) [1]. Due to the uncontrollable inflammatory reactions in the lung tissue, a large number of neutrophils, macrophages, and other inflammatory cells accumulate in the alveolus [27, 28]. The incidence of posttraumatic ARDS was significantly higher than that of other organs. Some studies have even reported that the probability of ARDS in trauma patients is 29% within 24 h, and on the fifth day after trauma, more than 90% of patients have ARDS [29]. In recent years, although great strides have been made in medicine, there was no change in the mortality rate from posttraumatic ARDS [8]. Early diagnosis, risk assessment and timely treatments in the initial periods of posttraumatic ARDS play a crucial role in reducing mortality [30]. The identification and development of new biomarkers can provide major insights into the pathophysiologic mechanisms underlying posttraumatic ARDS and can be helpful for the diagnosis, risk stratification and identification of candidate therapeutic targets [10, 31]. Bioinformatic analyses enable us to understand the molecular mechanisms of disease occurrence and development, providing a novel and effective way to identify potential diagnostic biomarkers and therapeutic targets as early-warning signals and for the timely treatment of posttraumatic ARDS [32]. In the course of this study, the hub genes were identified and analyzed to screen ten co-upregulated genes (GAPDH, MMP8, HGF, MAPK14, LCN2, CD163, ENO1, CD44, ARG1 and GADD45A) and five co-downregulated genes (HERC5, IFIT2, IFIT3, RSAD2 and IFIT1). Because the up-regulated DEGs are risk factors for the disease group, we have demonstrated the existing research results and related possible inferences of these ten genes one by one through previous literature research. First of all, GAPDH is well established as one of the molecules promoting apoptotic signaling in the cell nucleus. The role of GAPDH in regulating inflammation has been demonstrated by several studies [33, 34]. The damage to the heme chaperone caused by GAPDH nitrosylation leads to a decrease in catalase activity, which is a typical feature of the inflammatory process. GAPDH might also affect the inflammatory process through the regulation of tumor necrosis factor synthesis [35], with GAPDH-mediated proinflammatory cascades occurring after severe injury and sepsis [36, 37]. In such cases, blocking the inflammatory response is an important part of effective treatment. Therefore, in theory, GAPDH is a potential drug target. This speculation was confirmed in this study. This study provides a therapeutic target for the treatment of posttraumatic ARDS. Matrix metalloproteinases (MMPs) play major roles in cell differentiation, proliferation, wound healing, apoptosis and angiogenesis [38]. They also contribute to the pathogenesis of various diseases and conditions, such as inflammation, atherosclerosis and myocardial infarction [39, 40]. In previous decades, MMPs were considered to play only extracellular roles; however, this concept has been challenged in recent years. Further research is needed to clarify the functions of MMP8 within the cell. Understanding the biological functions of MMPs in cells is essential not only for understanding their physiological functions but also for discovering new therapeutic targets for the treatment of various pathologies. In identifying MMPs as a target for posttraumatic ARDS, this study suggests a new research direction [41]. HGF was first defined as the mitogenic protein of mature liver cells in 1984. It is a multifunctional cytokine that participates in cell morphogenesis, survival and proliferation and has anti-inflammatory effects [38, 39]. HGF seems to be related to secondary inflammation or anti-inflammatory effects, but the mechanism by which HGF regulates the immune response has not been resolved, and further research is needed. MAPK14, also known as cytokine inhibitory anti-inflammatory drug binding protein, is an osmotic regulator protein kinase that can be activated by exposure to many types of cellular stress. It plays a key role in triggering different disease states, such as inflammatory diseases, neurodegenerative diseases, cardiovascular diseases and cancer [42]. The MAPK14 pathway is closely related to many chronic inflammatory factors. These factors contribute to the production of proinflammatory cytokines and are essential in diseases such as Crohn's disease and chronic asthma [43, 44]. Some studies have shown that activated MAPK14 is highly expressed in the alveoli of smokers with chronic obstructive pulmonary disease (COPD). Therefore, inhibition of MAPK14 may be a valuable drug target for the treatment of COPD, and such inhibition for the treatment of ARDS, which involves the same pathological state of the respiratory system, also warrants study [45]. In addition, some studies have reported that MAPK14 is a valuable therapeutic target for acute or chronic inflammatory diseases [46]. LCN2, also known as neutrophil gelatinase-associated lipocalin, is a new type of adipocyte factor with 198 amino acids [47]. Several studies have shown that TNFα induces the expression and secretion of LCN2 [48], and lipopolysaccharide is a strong inducer of LCN2 expression in various tissues [49]. In addition, the expression of LCN2 mRNA in bronchial epithelium and type II lung cells has been found to be significantly increased in patients with lung inflammation [50]. These findings indicate that there is a direct or indirect link between LCN2 and posttraumatic ARDS. The expression of CD163 is upregulated in many diseases, but our understanding of the pathological role of this receptor in diseases seems incomplete [51]. CD163 binds to and degrades inflammatory cytokines, is a weak inducer of tumor necrosis factor-like cell apoptosis [52], and recognizes and mediates local immune responses to bacteria [53] and to internalize viruses [54]. Therefore, the macrophage scavenger receptor CD163, which is upregulated in many inflammatory and malignant diseases, is a promising target. As a part-time protein, ENO1 has a variety of biochemical functions. ENO1 catalyzes the conversion of 2-phosphoglycerate to phosphoenolpyruvate, which is an important step in glycolysis, and it plays an important role in multiple pathophysiological processes [55]. ENO1 binds to guanylate-binding protein and negatively regulates T cell signaling by interfering with early T cell receptor signaling [56]. Posttranslational modification activities are also important for the function of ENO1 in immunity [57, 58]. Inflammatory stimulation can induce the translocation of ENO1 from the cytoplasm to the cell membrane [59, 60]. Therefore, the conversion of ENO1 localization is related to inflammatory pathology, and its role in the pathology of posttraumatic ARDS warrants further study. The nonkinase transmembrane glycoprotein CD44 was first described as a lymphocyte homing receptor in 1983 and has attracted considerable interest recently. Generally, CD44 is widely expressed on vertebrate cells, and its ability to regulate tumor progression, metastasis, and disease prognosis has been extensively explored [61]. CD44 is a multifunctional transmembrane glycoprotein receptor that binds to hyaluronic acid. Extracellular and intracellular hyaluronic acid binds to CD44 and affects cell behavior. As a receptor, CD44 can trigger signal cascades that regulate cell functional properties, such as proliferation, migration, angiogenesis, and wound healing. The regulation of these pathways may be critical to the development of pathological conditions such as inflammation and cancer [62]. ARG1 is a cytoplasmic enzyme that is expressed in macrophages, bone marrow-derived suppressor cells, dendritic cells, and innate lymphoid group 2 cells in response to Th2-type cytokines (IL-4 and IL-13) [63, 64] and infection with pathogens related to other signaling factors [64]. In humans, ARG1 exists in the granulocyte granular compartment of healthy subjects [65], the peripheral blood mononuclear cells of patients after injury, and the activated monocytes of patients with autoimmune diseases. For patients with autoimmune imbalance after trauma, ARG1 has research value. GADD45A exhibits a variety of important functions in cells, including the inhibition of cell growth, the mediation of cell cycle arrest at G2/M, the induction of apoptosis, and interaction with p53, cyclin-dependent kinase 1 (CDK1), Cdc2 and cyclin B1 [66]. In summary, these 10 hub genes obtained through screening are theoretically related to posttraumatic ARDS. However, further research is needed to predict and treat the exact mechanisms underlying ARDS.

Our study has several limitations. The first limitation is that this study is exploratory in nature. Thus, further experimental studies and clinical trials should be carried out to obtain accurate verification and to validate our results. Another limitation to this research is that gene expression patterns are also dependent on important underlying comorbid conditions and can additionally be dependent on age. Specifically, the upregulated (or downregulated) genes are not specific to posttraumatic ARDS and may be upregulated (or downregulated) even more in certain patient populations, and/or the gene expression findings may be “driven” by cohorts of patients with certain chronic inflammatory processes or age-dependent inflammatory processes. The linkage of the acute and chronic inflammatory processes is imperative for further identifying the patients with “treatable traits” that would be most responsive and potentially least harmful to the treatment being tested in clinical trials. Because this study included information from a public dataset, secondary classification of subtypes could not be performed, which may result in inapplicability of the results to certain groups. Further clinical trials are needed to validate the subtypes. In addition, although the data analyzed represent RNA expression patterns within the first 4 days of a critical illness, the time course of the first days after onset of a critical illness, whether sepsis, ARDS or trauma, is often associated with a fluctuating inflammatory response. Therefore, future studies of similar RNA expression patterns should include more discrete time points for collecting RNA in relation to the onset of critical illness.

## Conclusion

In combination with data from previous studies and bioinformatic analyses, our study found that GAPDH, MMP8, HGF, MAPK14, LCN2, CD163, ENO1, CD44, ARG1, GADD45A, HERC5, IFIT2, IFIT3, RSAD2 and IFIT1 were related to the potential common mechanisms between severe injury trauma and ARDS. In addition, it can be seen from the results that the hub genes of trauma and ARDS are not the same as those of sepsis and ARDS, which on the other hand shows that posttraumatic ARDS has its own characteristic targets and is worthy of further exploration. These findings shed new light on the diagnosis of posttraumatic ARDS and identify candidate targets for therapeutic intervention. Further research will be needed to explore these possibilities.

## Data Availability

The open-access datasets are available through the following URL: https://www.ncbi.nlm.nih.gov/geo/query/acc.cgi?acc=GSE64711, https://www.ncbi.nlm.nih.gov/geo/query/acc.cgi?acc=GSE76293 and https://www.ncbi.nlm.nih.gov/geo/query/acc.cgi?acc=GSE100159. All data generated or analyzed during this study are available from the corresponding author on reasonable request.

## Abbreviations

ARDS: Acute respiratory distress syndrome
DEGs: Differentially expressed genes
GEO: Gene expression omnibus
ICU: Intensive care unit
GO: Gene ontology
KEGG: Kyoto encyclopedia of genes and genomes
CC: Cellular component
BP: Biological process
MF: Molecular function
PPI: Protein–protein interaction
GAPDH: Glyceraldehyde-3-phosphate dehydrogenase
MMP8: Matrix metallopeptidase 8
HGF: Hepatocyte growth factor
MAPK14: Mitogen-activated protein kinase 14
LCN2: Lipocalin 2
CD163: CD163 molecule
EN01: Enolase 1
CD44: CD44 molecule
ARG1: Arginase 1
GADD45A: Growth arrest and DNA-damage-inducible, alpha
HERC5: HECT and RLD domain containing E3 ubiquitin protein ligase 5
IFIT2: Interferon induced protein with tetratricopeptide repeats 2
IFIT3: Interferon induced protein with tetratricopeptide repeats 3
RSAD2: Radical S-adenosyl methionine domain containing 2
IFIT1: Interferon induced protein with tetratricopeptide repeats 1
